# Supplementary material for: The effects of task similarity during representation learning in brains and neural networks
Source: Nat Commun. 2025 Nov 29;16:10812. doi: 10.1038/s41467-025-66849-8 (PMC12669730; doi:10.1038/s41467-025-66849-8)
Supplement: Supplementary file 1 — Supplementary Information [file 41467_2025_66849_MOESM1_ESM.pdf]

The effects of task similarity during representation learning in brains and neural networks

N. Menghi[1*], W. J. Johnston[2], S. Vigano'[1,3], M. A. B. Hinrichs[1], B. Maess[1], S. Fusi[2,4], and C. F. Doeller[1,5]

[1]Max Planck Institute for Human Cognitive and Brain Sciences, Department of Psychology, Leipzig, Germany

[2]Center for Theoretical Neuroscience, Columbia University, New York, NY, USA Mortimer B. Zuckerman Mind, Brain and Behavior Institute, Columbia University, New York, NY, USA

[3]Center for Mind/Brain Sciences, University of Trento, Rovereto 38068, Italy

[4]Kavli Institute for Brain Sciences, Columbia University, New York, NY, USA

[5]Kavli Institute for Systems Neuroscience, Center for Neural Computation, The Egil and Pauline Braathen and Fred Kavli Center for Cortical Microcircuits, Jebsen Center for Alzheimer's Disease, Norwegian University of Science and Technology, Trondheim 7491, Norway

[*]Corresponding author

October 27, 2025

1 Supplementary Materials

1.1 Stimulus Mappings

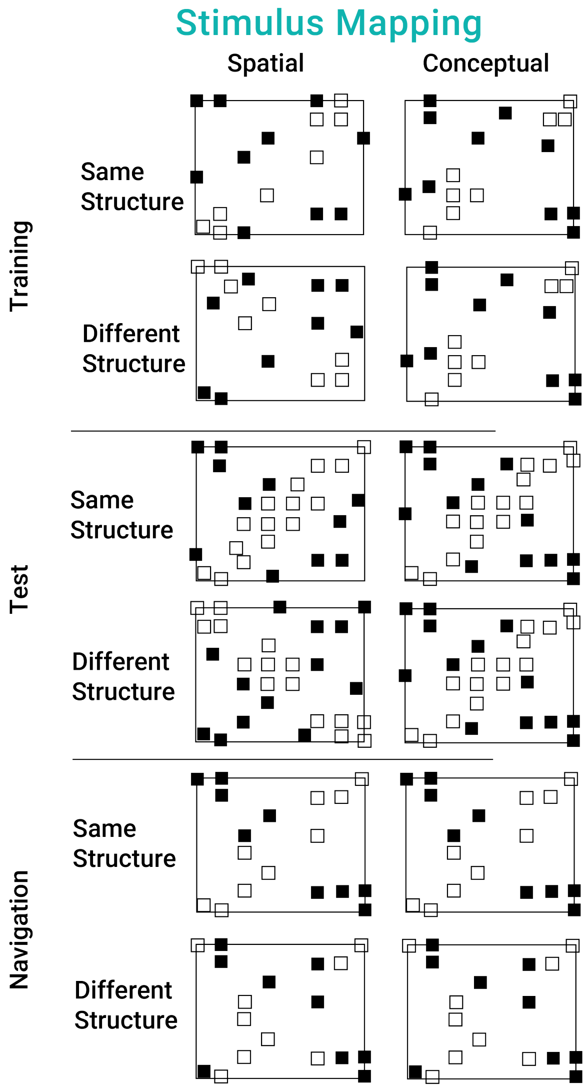

Supplementary Figure 1: Stimulus Mapping *The figure depicts the different configurations of conceptual or spatial features presented during each phase per task and group. Every configuration was repeated 8 times in both the training and the testing sessions for a total of 288 trials in the training and 448 per testing session. During the navigation phase, each starting configuration was repeated 10 times for a total of 180 trials.*

1.2 Behavioral Results

1.2.1 Learning Performance Testing Sessions

Accuracy was computed as the proportion of correct responses over all trials in each testing session and context (Spatial and Conceptual). We performed two between-subject t-tests to see if general performance in the two groups was different. We found that participants performance was not statistically different during the testing phases (First Testing Session: $t(52) = 1.489$, $p = 0.142$; Second Testing Session: $t(52) = 1.367$, $p = 0.177$).

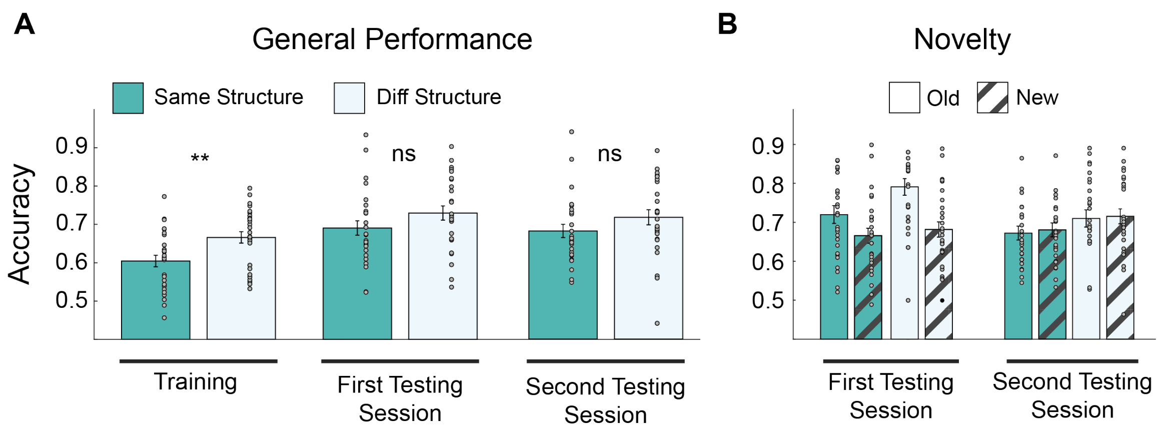

Supplementary Figure 2: General performance and Generalization ***Panel A*** *shows participants' accuracies during the training, pre-test and post-test phases. Participants are divided into SameSt (N=27) and DiffSt (N=27) group conditions. During training SameSt (mean = 0.6044, SD = 0.0783) and DiffSt (mean = 0.6661, SD = 0.0770) differed significantly, t(52) = –2.91, p = 0.005, Cohen's d = 0.80, 95% CI [–0.104, –0.019]. Asterisks indicate significance levels (*p < 0.05, **p < 0.01, ***p < 0.001).* ***Panel B*** *shows participants' performance for old and new stimuli during the testing phases. Participants are divided into SameSt and DiffSt following the color scheme of Panel A.*

1.2.2 Generalization Performance Testing Sessions

We then quantified generalization during the test session by computing the proportion of correct responses for novel configurations that, contrary to old ones, were not presented during training. We performed two 2x2 mixed-design ANOVAs, with between factor the group and within factor the novelty of the stimuli (new or old) on the first and second testing sessions. During the first testing session, we found a significant main effect of novelty ($F(1,52) = 44.655$, $p < 0.001$) and interaction ($F(1,52) = 5.025$, $p = 0.029$), indicating that participants performed better with old stimuli compared to new ones, but no group main effect ($F(1,52) = 2.663$, $p = 0.108$) (See Fig. 2B, Generalization panel). During the post-test, we found no significant difference (Group: $F(1,52) = 1.837$, $p = 0.181$; Novelty: $F(1,52) = 1.449$, $p = 0.234$; interaction: $F(1,52) = 0.059$, $p = 0.808$). These results indicate that participants' performance for novel stimuli, and thus their generalization, does not differ between the two group conditions (SameSt vs DiffSt). Albeit we do not find differences in generalization, participants' performance for the old stimuli differs only in the testing session after the training, but not in the last one.

1.2.3 Linear relationship between performance and decision boundary

To assess whether participants' accuracy was influenced by the decision boundary, we conducted a trial-wise analysis using a Generalized Linear Model (GLM). For each participant, we modeled accuracy (correct vs. incorrect response) as a function of the stimulus' Euclidean distance from the decision boundary and a bias (vector of only ones). Specifically, we fit such a regression model separately for the training and test phases. We then extracted, per participant, the beta coefficient and tested whether it was significantly greater than zero using a one-sample t-test. The results revealed that beta was significantly positive in both the training ($t(53)=8.213, p<0.001$) and test ($t(53)=17.146, p<0.001$) phases, indicating that accuracy improved as stimuli were further from the boundary. These findings suggest that participants were sensitive to the underlying task structure, with errors occurring more frequently near the decision boundary. This pattern remained stable across training and test, further supporting the idea that participants developed a structured

representation rather than relying on a simple heuristic.

1.3 MEG Results

Fig. 3 shows the correlation between the neural dissimilarity matrices of the sensors belonging to the significant clusters and the Stimulus-bound model averaged across all stimulus configurations during the training phase per context.

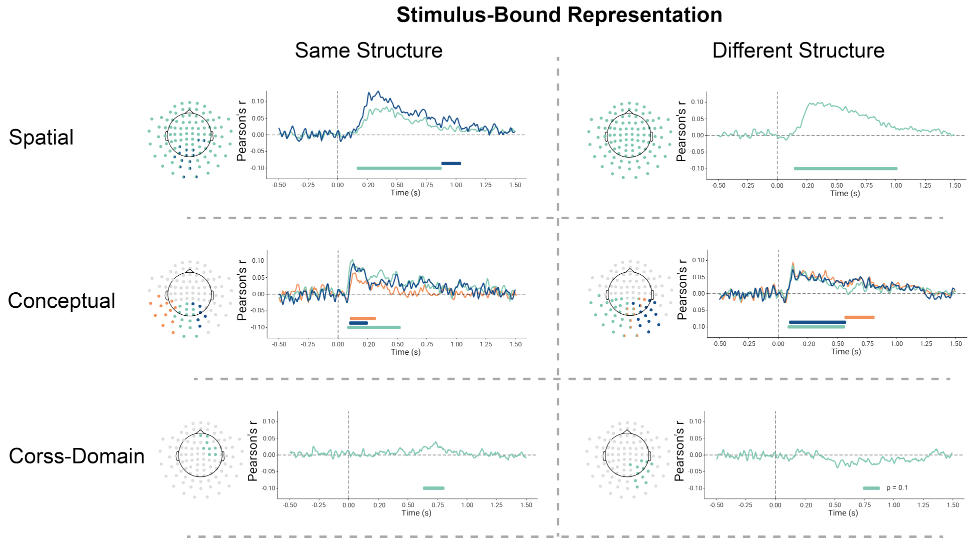

Supplementary Figure 3: Stimulus-bound Representation during training *Each panel shows the results of the cluster-permutation correction of the correlation between stimulus-bound models and the neural dissimilarity matrix. Clusters are color-coded so that significant sensors, their significant time course and correlation are shown. Each cluster is associated with one colour. Results are divided into spatial, conceptual and cross-domain spaces for both the SameSt and DiffSt groups.*

Fig. 4 shows the correlation between the neural dissimilarity matrices of the sensors of the significant clusters and the task-relevant model averaged across all stimulus configurations during the training phase per context.

Fig. 5 shows the correlation between the neural dissimilarity matrices of the sensors of the significant clusters and the Stimulus-bound model averaged across all stimulus configurations per context during both testing phases.

Fig. 6 shows the correlation between the neural dissimilarity matrices of the sensors of the significant clusters and the task-relevant model averaged across all stimulus configurations per context during both testing phases.

1.3.1 Wholebrain RSA

In our time-resolved RSA analysis across sensors, we examined how the representational structure of neural activity evolves over time in response to task stimuli. For each time point, we extracted multivariate patterns of MEG sensor activity across all sensors and computed a neural dissimilarity matrix (RDM) based on pairwise dissimilarities (using pearson correlational distance) between conditions. This process was repeated independently for each time point in the peri-stimulus window, producing a temporal sequence of neural RDMs. Each neural RDM was then compared—via Spearman correlation—with model RDMs capturing hypothesized relationships between spatial,

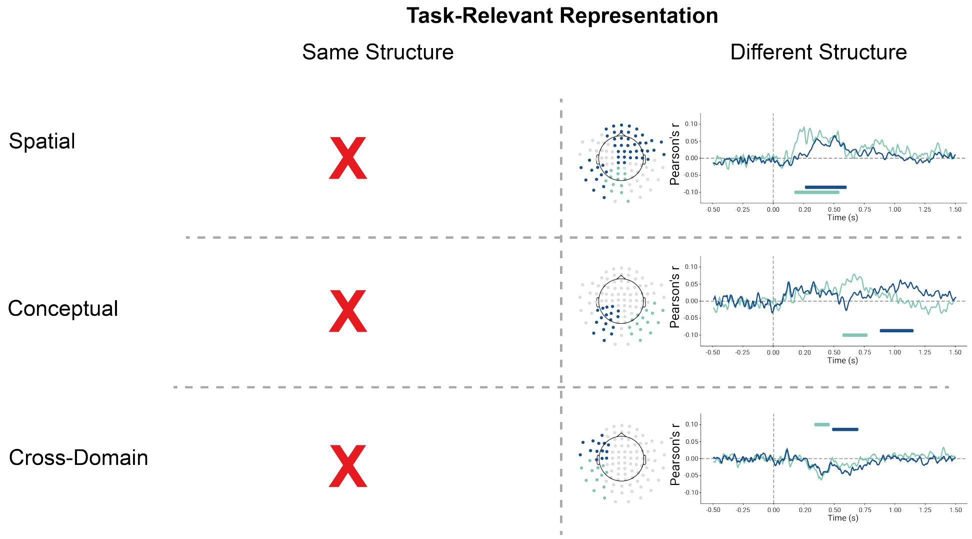

Supplementary Figure 4: Task-Relevant Representation during training *Each panel shows the results of the cluster-permutation correction of the correlation between stimulus-bound models and the neural dissimilarity matrix. X's indicate no significant clusters were found. Clusters are color-coded so that significant sensors, their significant time course and correlation are shown. Each cluster is associated with one colour. Results are divided into spatial, conceptual and cross-domain spaces for both the SameSt and DiffSt groups.*

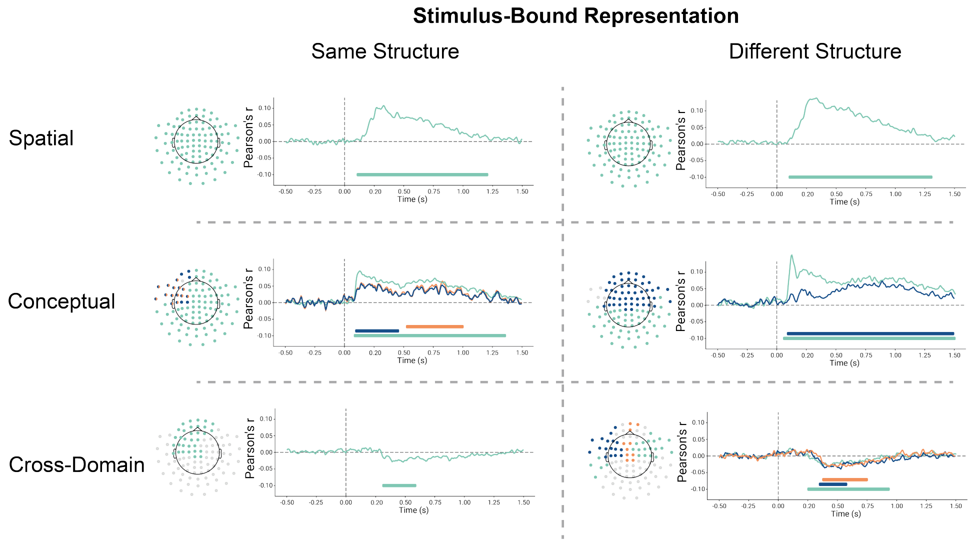

Supplementary Figure 5: Stimulus-bound Representation during test *Each panel shows the results of the cluster-permutation correction of the correlation between stimulus-bound models and the neural dissimilarity matrix. Clusters are color-coded so that significant sensors, their significant time course and correlation are shown. Each cluster is associated with one colour. Results are divided into spatial, conceptual and cross-domain spaces for both the SameSt and DiffSt groups.*

conceptual and cross-domain conditions in both stimulus-bound and task-relevant spaces. This yielded a time course of correlation values reflecting how strongly the neural data at each time point aligned with the model's representational structure. The results were then cluster corrected with the same procedure described in the main text.

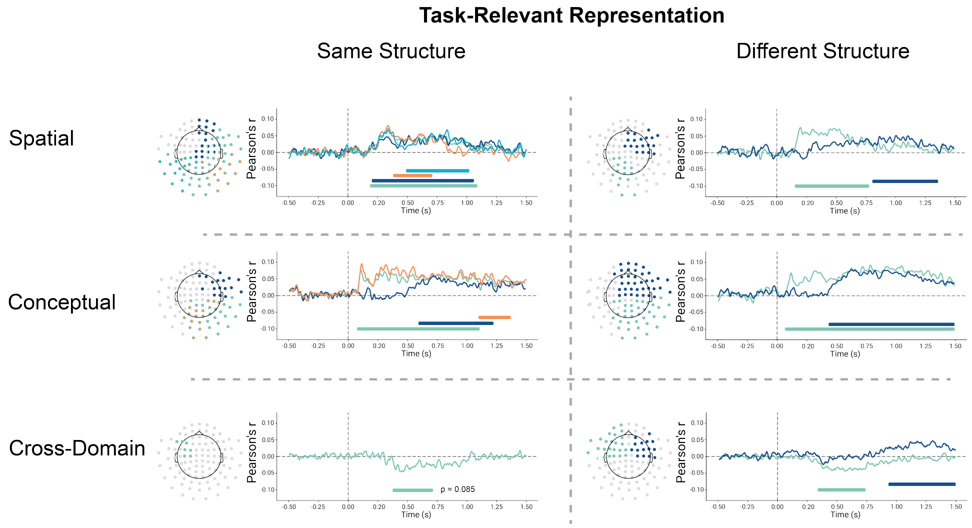

Supplementary Figure 6: Task-Relevant Representation during test *Each panel shows the results of the cluster-permutation correction of the correlation between stimulus-bound models and the neural dissimilarity matrix. Clusters are color-coded so that significant sensors, their significant time course and correlation are shown. Each cluster is associated with one colour. Results are divided into spatial, conceptual and cross-domain spaces for both the SameSt and DiffSt groups.*

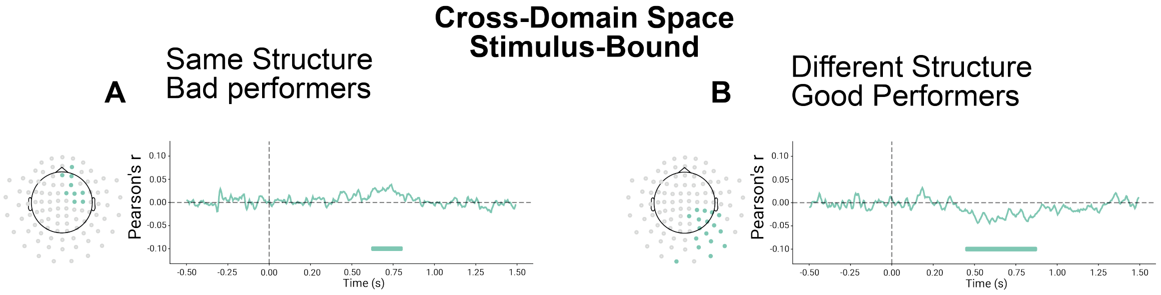

Supplementary Figure 7: We divided participants into good and bad performers according to median split. We then computed neural similarity between stimulus-bound representations of the stimuli during the training phase and the cross-domains model. We found a positive significant cluster in the bad performers in the SameSt group and a negative cluster in the good performers in the DiffSt group. **Panel A** *shows the results of the cluster-permutation correction of stimulus-bound representations for cross-domain space during training for participants in the SameSt group. Clusters are colour-coded so that significant sensors, their significant time course and correlation are shown. Each cluster is associated with each colour. Results are divided into spatial, conceptual and cross-domain spaces for both the SameSt and DiffSt groups.* **Panel B** *shows the results of the cluster-permutation correction of the stimulus-bound representations for cross-domain space during training for participants in the DiffSt group. Clusters are colour-coded so that significant sensors, their significant time course and correlation are shown. Each cluster is associated with one colour.*

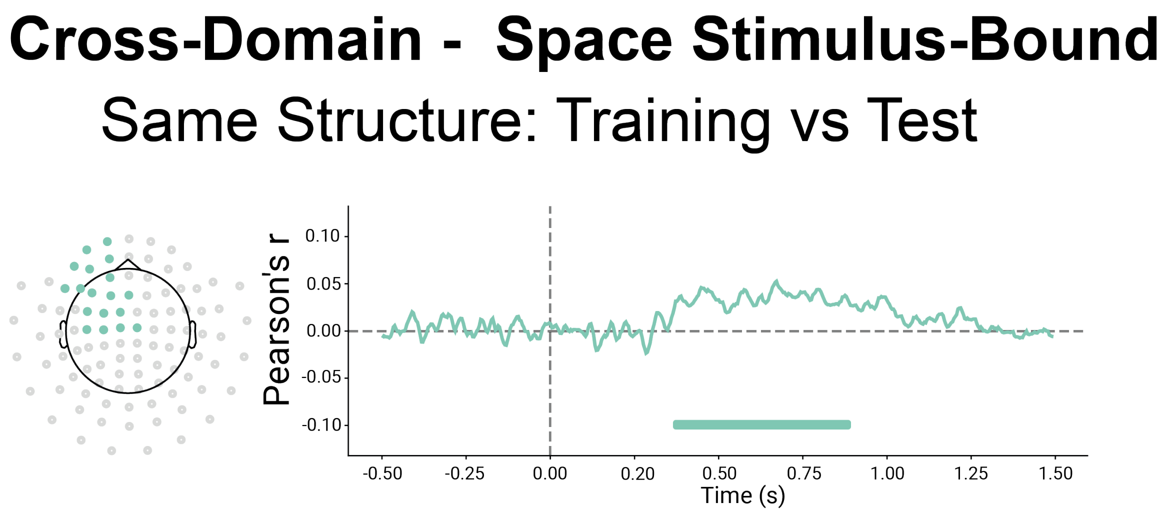

Supplementary Figure 8: We tested the neural similarity between stimulus-bound representations of the stimuli during the training phase compared to the test phase. We found only a positive significant cluster in the bad performers in the SameSt group. *The figure shows the results of the cluster-permutation correction of stimulus-bound representations for cross-domain space comparing the training phase with testing one in the SameSt group. Clusters are colour-coded so that significant sensors, their significant time course and correlation are shown. Each cluster is associated with each colour. Results are divided into spatial, conceptual and cross-domain spaces for both the SameSt and DiffSt groups.*

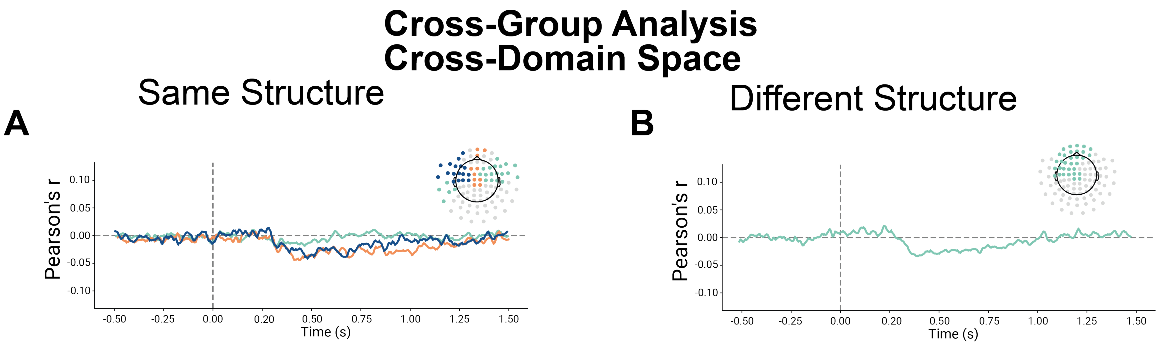

Supplementary Figure 9: Cross-group validation of significant clusters in MEG data for the cross-domain space. *Significant clusters (channels and time points) identified in the SameSt group were tested in the DiffSt group, and vice versa. (A) Three significant clusters were identified in the DiffSt group and tested in the SameSt group. The fronto-central and fronto-left lateral clusters showed the same significant pattern ($t(26) = -2.77$, $p = 0.01$; $t(26) = -2.82$, $p = 0.008$), while the fronto-right lateral cluster was not significant ($t(26) = -0.56$, $p = 0.579$). (B) The significant cluster from the SameSt group exhibited the same negative trend when tested in the DiffSt group ($t(26) = -4.57$, p ¡ 0.001).*

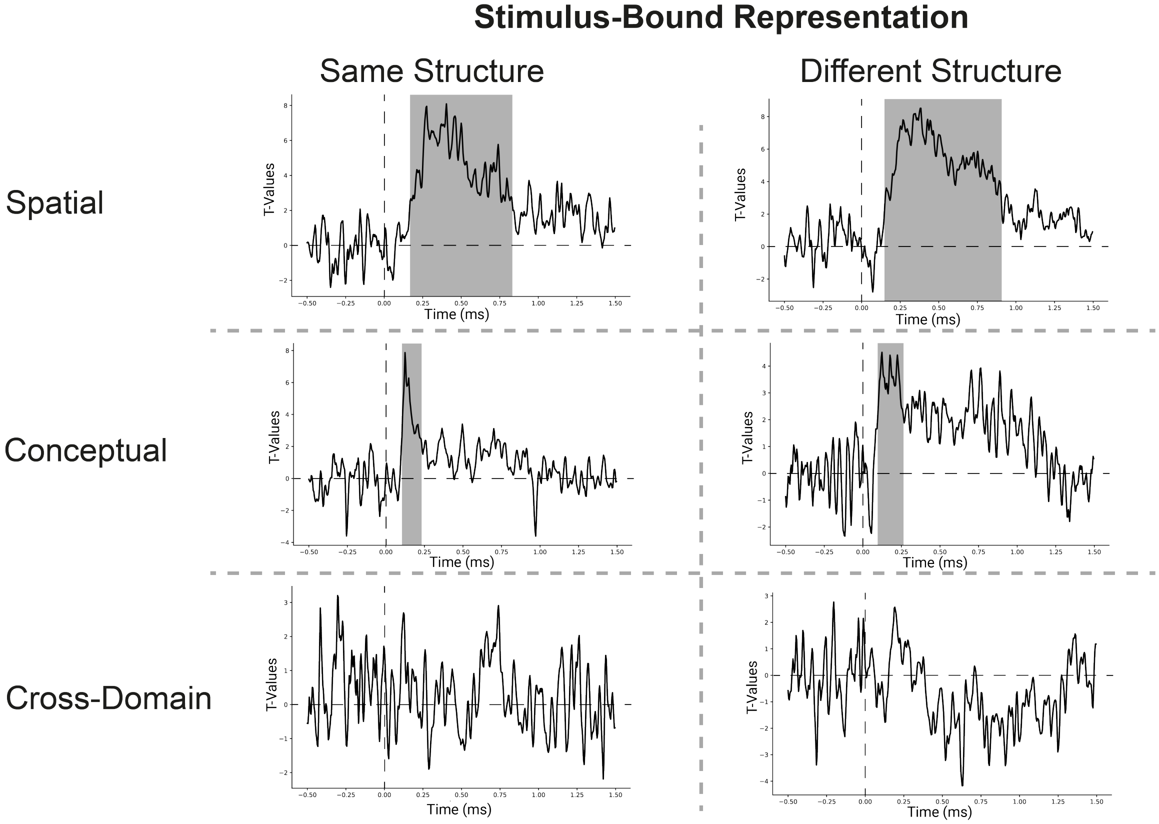

Supplementary Figure 10: Time-resolved RSA results during training for stimulus-bound spaces. *This figure shows the time course of representational similarity analysis (RSA) results during the training phase, separately for the spatial, conceptual, and cross-domain model RDMs in the Same Structure (SameSt) and Different Structure (DiffSt) groups. The x-axis indicates time relative to stimulus onset (in milliseconds), and the y-axis shows the corresponding t-values from second-level statistics comparing model-brain correlations across participants. Each panel displays the time-resolved correlation between the neural dissimilarity matrix (computed across all MEG sensors) and the model dissimilarity matrix for a specific representational space. Grey shaded regions indicate time windows in which cluster-based permutation tests revealed significant clusters (p ¡ 0.05, two-tailed). These results reflect how the neural activity during training aligns with the spatial, conceptual, and cross-domain stimulus-bound structure across time in each group.*

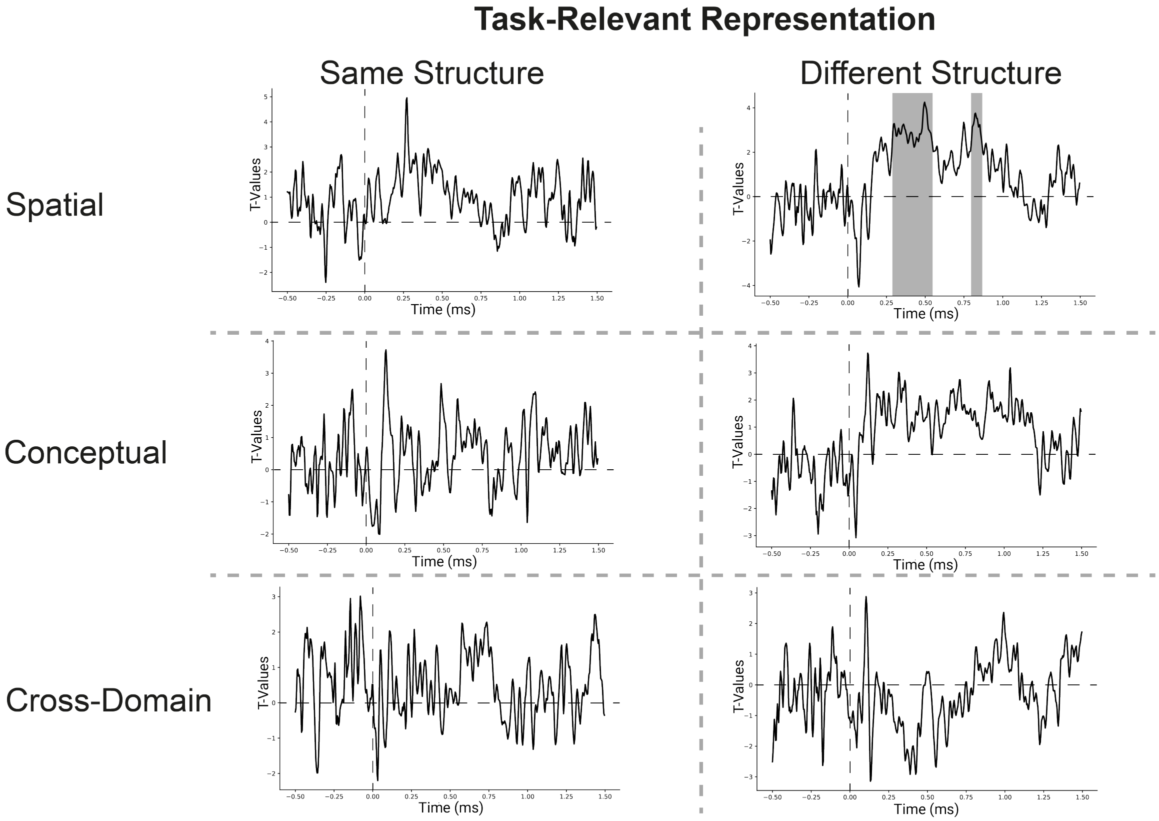

Supplementary Figure 11: Time-resolved RSA results during training for task-relevant spaces. *This figure shows the time course of representational similarity analysis (RSA) results during the training phase, separately for the spatial, conceptual, and cross-domain model RDMs in the Same Structure (SameSt) and Different Structure (DiffSt) groups. The x-axis indicates time relative to stimulus onset (in milliseconds), and the y-axis shows the corresponding t-values from second-level statistics comparing model-brain correlations across participants. Each panel displays the time-resolved correlation between the neural dissimilarity matrix (computed across all MEG sensors) and the model dissimilarity matrix for a specific representational space. Grey shaded regions indicate time windows in which cluster-based permutation tests revealed significant clusters (p ¡ 0.05, two-tailed). These results reflect how the neural activity during training aligns with the spatial, conceptual, and cross-domain stimulus-bound structure across time in each group.*

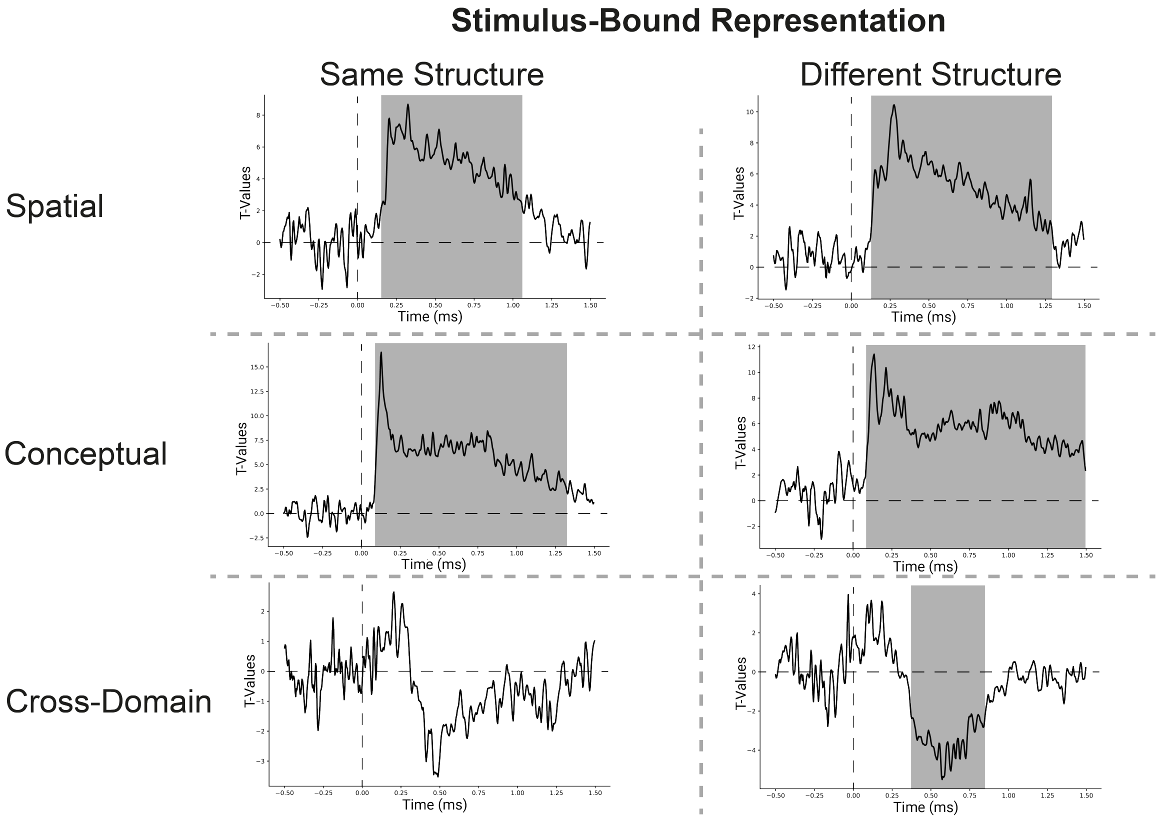

Supplementary Figure 12: Time-resolved RSA results during testing for stimulus-bound spaces. *This figure shows the time course of representational similarity analysis (RSA) results during the testing phase, separately for the spatial, conceptual, and cross-domain model RDMs in the Same Structure (SameSt) and Different Structure (DiffSt) groups. The x-axis indicates time relative to stimulus onset (in milliseconds), and the y-axis shows the corresponding t-values from second-level statistics comparing model-brain correlations across participants. Each panel displays the time-resolved correlation between the neural dissimilarity matrix (computed across all MEG sensors) and the model dissimilarity matrix for a specific representational space. Grey shaded regions indicate time windows in which cluster-based permutation tests revealed significant clusters (p ¡ 0.05, two-tailed). These results reflect how the neural activity during training aligns with the spatial, conceptual, and cross-domain stimulus-bound structure across time in each group.*

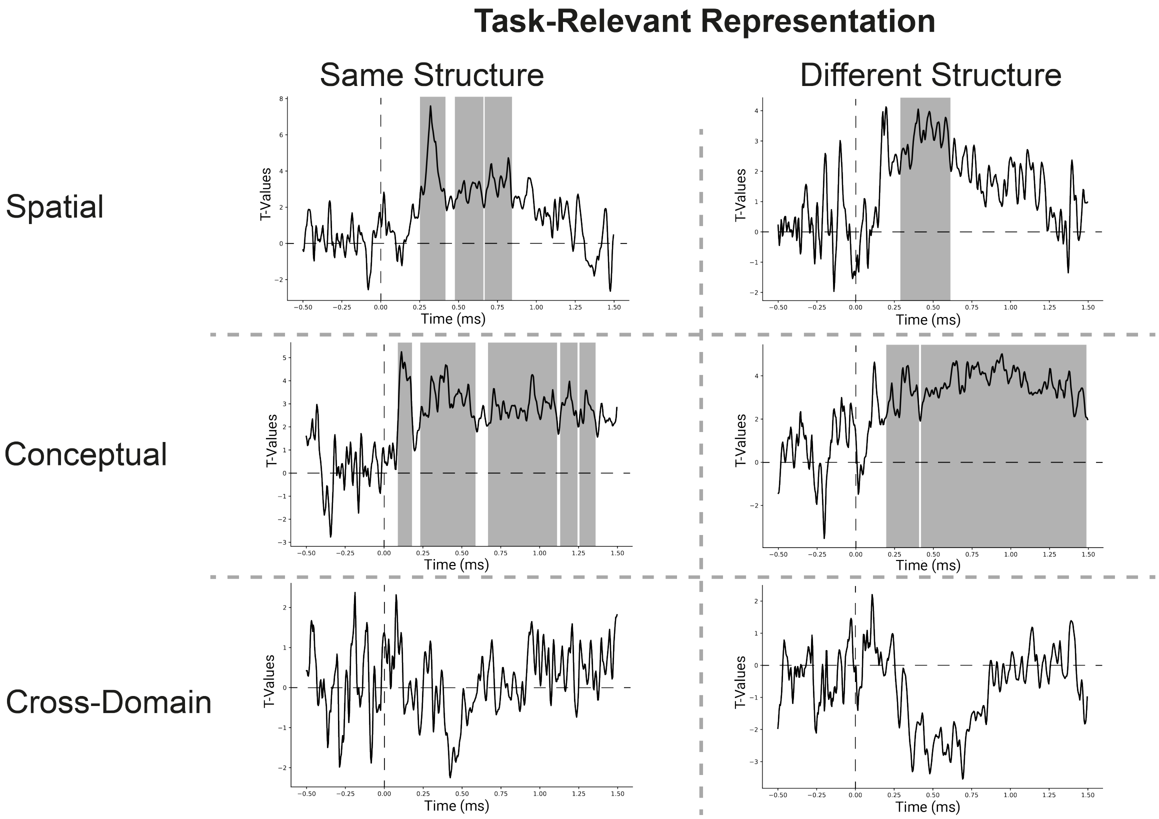

Supplementary Figure 13: Time-resolved RSA results during testing for task-relevant spaces. *This figure shows the time course of representational similarity analysis (RSA) results during the testing phase, separately for the spatial, conceptual, and cross-domain model RDMs in the Same Structure (SameSt) and Different Structure (DiffSt) groups. The x-axis indicates time relative to stimulus onset (in milliseconds), and the y-axis shows the corresponding t-values from second-level statistics comparing model-brain correlations across participants. Each panel displays the time-resolved correlation between the neural dissimilarity matrix (computed across all MEG sensors) and the model dissimilarity matrix for a specific representational space. Grey shaded regions indicate time windows in which cluster-based permutation tests revealed significant clusters (p ¡ 0.05, two-tailed). These results reflect how the neural activity during training aligns with the spatial, conceptual, and cross-domain stimulus-bound structure across time in each group.*
