# Supplementary material for: The effects of task similarity during representation learning in brains and neural networks
Source: Nat Commun. 2025 Nov 29;16:10812. doi: 10.1038/s41467-025-66849-8 (PMC12669730; doi:10.1038/s41467-025-66849-8)
Supplement: Supplementary file 2 — Reporting Summary [file 41467_2025_66849_MOESM2_ESM.pdf]

nature portfolio

Corresponding author(s): Nicholas Menghi

Last updated by author(s): 2025/10/14

Reporting Summary

Nature Portfolio wishes to improve the reproducibility of the work that we publish. This form provides structure for consistency and transparency in reporting. For further information on Nature Portfolio policies, see our Editorial Policies and the Editorial Policy Checklist.

Statistics

For all statistical analyses, confirm that the following items are present in the figure legend, table legend, main text, or Methods section.

n/a	Confirmed	
☐	☒	The exact sample size (*n*) for each experimental group/condition, given as a discrete number and unit of measurement
☐	☒	A statement on whether measurements were taken from distinct samples or whether the same sample was measured repeatedly
☐	☒	The statistical test(s) used AND whether they are one- or two-sided<br>*Only common tests should be described solely by name; describe more complex techniques in the Methods section.*
☐	☒	A description of all covariates tested
☐	☒	A description of any assumptions or corrections, such as tests of normality and adjustment for multiple comparisons
☐	☒	A full description of the statistical parameters including central tendency (e.g. means) or other basic estimates (e.g. regression coefficient) AND variation (e.g. standard deviation) or associated estimates of uncertainty (e.g. confidence intervals)
☐	☒	For null hypothesis testing, the test statistic (e.g. *F*, *t*, *r*) with confidence intervals, effect sizes, degrees of freedom and *P* value noted<br>*Give P values as exact values whenever suitable.*
☒	☐	For Bayesian analysis, information on the choice of priors and Markov chain Monte Carlo settings
☒	☐	For hierarchical and complex designs, identification of the appropriate level for tests and full reporting of outcomes
☐	☒	Estimates of effect sizes (e.g. Cohen's *d*, Pearson's *r*), indicating how they were calculated

Our web collection on statistics for biologists contains articles on many of the points above.

Software and code

Policy information about availability of computer code

Data collection	Psychtoolbox and Matlab were used to collect data
Data analysis	a combination of Matlab, Python and MNE python

For manuscripts utilizing custom algorithms or software that are central to the research but not yet described in published literature, software must be made available to editors and reviewers. We strongly encourage code deposition in a community repository (e.g. GitHub). See the Nature Portfolio guidelines for submitting code & software for further information.

Data

Policy information about availability of data

All manuscripts must include a data availability statement. This statement should provide the following information, where applicable:

- Accession codes, unique identifiers, or web links for publicly available datasets
- A description of any restrictions on data availability
- For clinical datasets or third party data, please ensure that the statement adheres to our policy

Raw data are protected and are not available due to data privacy. Preprocessed data will be made available upon request to the corresponding author. Processed data and source data are provided with this paper and available at https://doi.org/10.6084/m9.figshare.30353515.

Research involving human participants, their data, or biological material

Policy information about studies with human participants or human data. See also policy information about sex, gender (identity/presentation), and sexual orientation and race, ethnicity and racism.

Reporting on sex and gender	Participants included 27 males and 33 females; however, no hypotheses were formulated regarding potential differences based on sex or gender.
Reporting on race, ethnicity, or other socially relevant groupings	Data on race, ethnicity, or other socially relevant groupings were not collected for this study.
Population characteristics	See above
Recruitment	Participants were recruited from a database of volunteers.
Ethics oversight	University of Leipzig

Note that full information on the approval of the study protocol must also be provided in the manuscript.

Field-specific reporting

Please select the one below that is the best fit for your research. If you are not sure, read the appropriate sections before making your selection.

☐ Life sciences ☒ Behavioural & social sciences ☐ Ecological, evolutionary & environmental sciences

For a reference copy of the document with all sections, see nature.com/documents/nr-reporting-summary-flat.pdf

Behavioural & social sciences study design

All studies must disclose on these points even when the disclosure is negative.

Study description	Participants were trained to perform an associative learning task across two contexts, predicting whether a seed would 'grow' or 'die' based on distinct sets of features determined by the seed's identity. One context required learning associations involving 'conceptual features' (opacity of sun and water images), while the other involved 'spatial features' (seed's planting position). Participants were assigned to either a 'Same structure' group, with consistent feature-outcome mappings across contexts, or a 'Different structure' group, with flipped mappings. Quantitative data were collected during training via feedback learning and subsequent testing, conducted in two sessions on the same day, separated by an unrelated navigation task.
Research sample	A total of 60 volunteers participated in the experiment (mean age = 27.91, SD = 4.21, 27 males, 33 females
Sampling strategy	Participants were randomly sampled from a volunteer database, and the sample size was determined based on prior studies.
Data collection	Participants completed a MEG experiments inside a sound-attenuated, dimly lit, and magnetically shielded room. Stimuli were displayed on a rear-projection screen with a spatial resolution of 800x600 pixels and a refresh rate of 60 Hz using the Psychophysics Toolbox for Matlab (Mathworks). Each participant was trained to do two computerised tasks in an interleaved fashion.
Timing	Data collection started in May 2022 and finished in September 2023
Data exclusions	Data from six participants were discarded because their performance was below the chance level (56\%) in both the spatial and the conceptual tasks.
Non-participation	No participants dropped out.
Randomization	Participants were randomly assigned to either a "Same structure" (SameSt) or a "Different structure" group (DiffSt), with 30 participants in each group (27 per group after discarding bad performers).

Reporting for specific materials, systems and methods

We require information from authors about some types of materials, experimental systems and methods used in many studies. Here, indicate whether each material, system or method listed is relevant to your study. If you are not sure if a list item applies to your research, read the appropriate section before selecting a response.

Materials & experimental systems

n/a	Involved in the study	
☒	☐	Antibodies
☒	☐	Eukaryotic cell lines
☒	☐	Palaeontology and archaeology
☒	☐	Animals and other organisms
☒	☐	Clinical data
☒	☐	Dual use research of concern
☒	☐	Plants

Methods

n/a	Involved in the study	
☒	☐	ChIP-seq
☒	☐	Flow cytometry
☒	☐	MRI-based neuroimaging

Plants

Seed stocks	n/a
Novel plant genotypes	n/a
Authentication	n/a
